# Case report: Kaposi hemangioendothelioma of the right upper limb with the Kasabach–Merritt phenomenon: A potentially lethal diagnostic challenge

**DOI:** 10.3389/fped.2022.995399

**Published:** 2022-11-01

**Authors:** Levin Belani, Jamari Sapuan, Shalimar Abdullah, Erica Yee Hing, C-Khai Loh, Hamidah Alias

**Affiliations:** ^1^ Department of Orthopaedic and Traumatology, UKM Medical Centre, Faculty of Medicine, Universiti Kebangsaan Malaysia, Kuala Lumpur, Malaysia; ^2^ Department of Radiology, UKM Medical Centre, Faculty of Medicine, Universiti Kebangsaan Malaysia, Kuala Lumpur, Malaysia; ^3^ Pediatric Hematology and Oncology Unit, Department of Pediatrics, UKM Medical Centre, Faculty of Medicine, Universiti Kebangsaan Malaysia, Kuala Lumpur, Malaysia

**Keywords:** kaposi hemangioendothelioma, Kasabach–Merritt, upper limb, sirolimus, treatment outcome

## Abstract

Kaposi hemangioendothelioma (KHE) is a rare vascular neoplasm that presents usually within the first year of life. Because of its rarity and complexity, there is often a delay in diagnosis. KHE could be associated with a life-threatening consumptive coagulopathy named the Kasabach–Merritt phenomenon (KMP). Here, we present the case of a 2-month-old girl who presented with progressive redness and swelling of her right upper limb over 6 weeks. Multiple health practitioners misdiagnosed her condition as an insect bite, cellulitis, and necrotizing fasciitis and gave treatment accordingly, which proved futile. A full blood count revealed bicytopenia of anemia and thrombocytopenia, a normal coagulation cascade, low fibrinogen, and raised D-Dimer levels. The imaging was suggestive of a high-flow vascular tumor likely to be a KHE. Subsequently, she was started on single-agent oral sirolimus with a dose increment to achieve satisfactory therapeutic levels and was treated for 1 year. She successfully completed the treatment regimen and had only transient hypertriglyceridemia, which resolved upon the completion of treatment. Currently, she is in remission 3 years after treatment. Keeping her case as an example, we would like to highlight the potentially lethal misdiagnosis of KHE with KMP, the importance of an early diagnosis of this condition, and the successful treatment outcome with single-agent sirolimus.

## Introduction

Kaposi hemangioendothelioma (KHE) is a rare vascular neoplasm diagnosed during infancy or early childhood with a prevalence and incidence of 0.91 and 0.071 per 100,000 children, respectively (1). Men have a slight predominance over women, as reported in two large retrospective studies (2, 3). KHE is mostly evident (90%) within the first year of life (4). The etiology of this disease remains unknown, and in most cases, KHE occurs insidiously (5). The Kasabach–Merritt phenomenon (KMP) is a life-threatening coagulopathy occurring in 70% of KHE cases (1). In view of the rarity of KHE with KMP, delay in diagnosis may lead to morbidity and mortality of up to 30% mostly due to life-threatening hemorrhage, cardiac failure, and local invasion of the neoplasm (6).

## Case presentation

A 2-month-old girl presented with a 6-week history of progressive swelling over the right forearm. She was delivered at term *via* emergency lower-section cesarean section with a birth weight of 2.92 kg. Antenatally, the mother had gestational diabetes mellitus and had a history of threatened preterm labor. The intrapartum and postpartum periods were uneventful. At 2 weeks old, a small erythema was noted at the lateral right forearm that progressed to involve the entire right forearm, with progressive swelling in the next 3 weeks. There was no history of trauma or fall. There was no family history of malignancy. The child remained active, was feeding well, was not fretful, and was afebrile. There was no limitation to her range of motion, nor did she cry upon handling.

At 1 week of illness, her mother brought her to a general practitioner, and she was treated symptomatically for urticaria, presumably caused by an insect bite. However, as the redness in her upper limb persisted and the swelling enlarged, she was brought to another medical practitioner and was then admitted to a hospital a week later and treated for cellulitis of the right upper limb with intravenous antibiotics. However, a radiograph of the affected upper limb findings was found to be unremarkable. Clinically, the girl remained afebrile, active, and thriving, and did not have any limitation of her right upper limb range of motion. Despite treatment with two courses of antibiotics, no signs of improvement were observed. Then, ultrasound imaging and magnetic resonant imaging (MRI) of the right upper limb were performed, and subsequently, the private orthopedic surgeon referred the girl to our center for further management. Following multiple investigations by a multidisciplinary team, a definitive diagnosis was arrived at.

**Figure 1:**
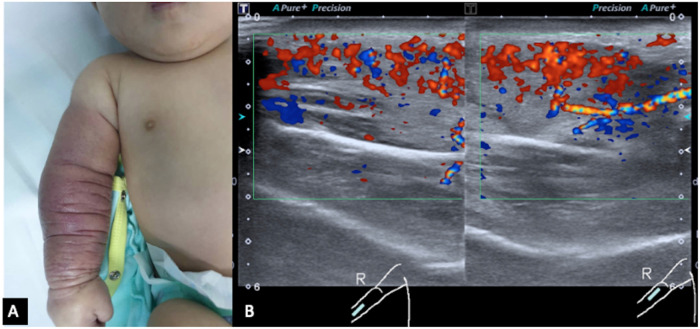
(**A**) Red discoloration and swelling of the right forearm up to the mid-arm. Redness and swelling are circumferential throughout the forearm and mid-arm. (**B**) Ultrasound color Doppler of the right forearm in longitudinal view shows marked hypervascularity in the swollen subcutaneous layer and muscles. Both arterial and venous spectral waveforms are detected. There is no margin seen bordering the lesion. These findings are suggestive of a high-flow vascular tumor.

She was initially referred for soft tissue infection of the right upper limb with a differential of necrotizing fasciitis. The resident orthopedic team assessed the child at the age of 2 months. A clinical examination revealed a huge discrete swelling of the right upper limb extending from her right wrist up to the mid-arm and was associated with red-bluish discoloration, which was dry, cool, and non-tender (Figure 1A). The range of motion of her elbow was 0–120° and her neurovascular status of the limb was intact and normal. She was active and afebrile.

**Figure 2:**
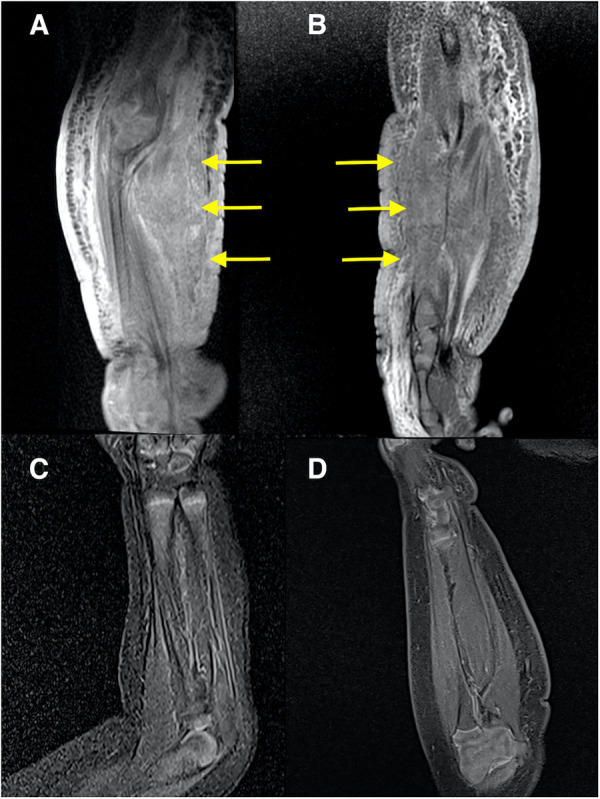
MRI of the right forearm: (**A,B**) at diagnosis; (**A**) coronal T1 fat-saturated post-contrast; (**B**) sagittal PD fat-saturated. Both images show a diffuse infiltrative abnormal signal and thickening of the skin, subcutaneous layer, and muscles of the forearm without a clear margin (arrows). The subcutaneous oedema extends into the arm. Moderate enhancement is seen in the post-contrast sequence (**A**). MRI of the right forearm: (**C,D**) at 6 months after treatment started; (**C**) Coronal STIR; (**D**) Sagittal T1 fat-saturated post-contrast. Both images show a resolution of the abnormal changes.

Initial blood tests revealed that the child had bicytopenia. Her hemoglobin level was 6.2 g/dl with a platelet count of 8 × 10^9^/L. Further investigations were suggestive of consumptive coagulopathy with a fibrinogen level of 1 g/L and a D-Dimer level of 11.5 μg/ml. Her coagulation profile was normal with a PT of 13.8 s, APTT of 43.6 s, and INR of 1.08. The blood investigation results were not suggestive of bacterial infection. Subsequently, she was referred to the pediatric hematology team and a clinical diagnosis of Kaposi hemangioendothelioma with the Kasabach–Merritt phenomenon (KMP) was made. The MRI of the right forearm demonstrated a diffuse infiltrative lesion that crossed multiple planes in the forearm without a clear margin (Figures 2A,B), and the ultrasound doppler confirmed the lesion to be a high-flow vascular tumor (Figure 1B).

The infant received packed red blood cells, platelet, and fresh frozen plasma transfusions during admission as she bled from the intravenous line insertion site. Single-agent oral sirolimus was commenced with an initial dosage of 0.2 mg BD (0.8 mg/m^2^/dose). According to the institutional policy, sirolimus can be used only for children aged 2 years and above; however, ethical approval was obtained for the drug to be used in this infant. After 14 days of sirolimus treatment, a reduction in redness and swelling of the right upper limb was observed and the platelet normalized to 123 × 10^9^/L. The patient was discharged home after 1 week of admission, and oral sirolimus of the same dose was continued for 4 months before the dose was increased to 0.4 mg BD, 0.6 mg BD, and then 0.8 mg BD up to a total duration of 1 year. There was an issue related to compliance with sirolimus during months 3 and 4 of the treatment. During treatment, the serum sirolimus levels were closely monitored to achieve a trough level of between 10 and 15 ng/ml. The serum levels were checked weekly during the first month of treatment, then biweekly for the subsequent month, and then monthly. After a stable dose was achieved, therapeutic drug monitoring was performed every 3 months. A prophylaxis for pneumocystis pneumonia, trimethoprim/sulfamethoxazole, was started concomitantly until the treatment was completed. An ultrasound-guided biopsy was performed under sedation 2 months after treatment started with a normal coagulation profile, and the histopathological examination showed a proliferation of multiple scattered, small, round, and some slit-like vascular channels within the deep dermis and subcutaneous fat that are lined by bland endothelial cells. Some of the cells appeared to form a glomeruloid structure. The endothelial cells were positive for CD31 and CD34, which were suggestive of a vascular lesion favoring KHE.

**Figure 3:**
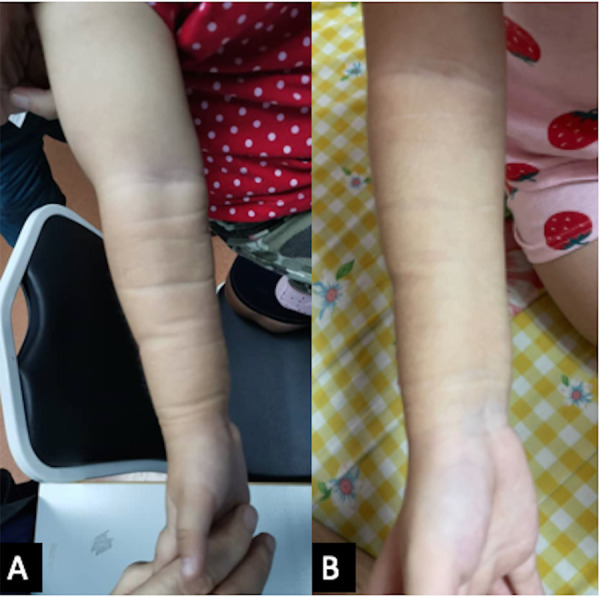
(**A**) The right upper limb shows a resolution of discoloration and swelling after 1 year of oral sirolimus. (**B**) The girl’s current photograph of her right upper limb at 3 years of age.

She was also followed up regularly following the treatment protocol to monitor the full blood count, renal profile, liver function, and fasting serum lipids. She had transient hypertriglyceridemia, which resolved after the treatment was completed. A repeat MRI after 6 months of treatment revealed a complete resolution of the lesion (Figures 2C,D); however, clinically, there was some residual skin discoloration. After the completion of the dosage of 1 year of oral sirolimus, the right upper limb swelling and discoloration resolved completely (Figure 3A). Currently, at the age of 3 years, she remains in remission (Figure 3B). Her parents are highly satisfied with the treatment outcome and are compliant with the follow-up regime.

## Discussion

A right upper limb redness and swelling in infants is often diagnosed as cellulitis, necrotizing fasciitis, hematoma, or a vascular tumor. The baby girl who was referred to our center initially presented herself to multiple health practitioners, who misdiagnosed her condition and delayed the definitive diagnosis of KHE with KMP, which could potentially be fatal. The delay in diagnosis could be explained by the rarity of the disease condition, a lack of experience in clinical diagnosis, the deep location of the tumor (mediastinum or retroperitoneum), and coagulopathy, making biopsy challenging (7).

The clinical diagnosis of KHE requires a thorough history and examination, blood investigations, imaging, and biopsy. KHE presentation could be heterogenous and variable, thus providing a diagnostic challenge. The spectrum of presentation could vary from cutaneous lesions with variable appearances to deep masses without any cutaneous signs. Patients with KMP and without KMP could also be distinguished through examination (5). KHE presents commonly with a single soft tissue mass with cutaneous findings, which range from an erythematous papule or nodule to a firm purplish tumor (5). These lesions are usually painful, hot to the touch, swollen, and purpuric in patients with KMP (5). In this case, the initial presentation to the orthopedic team did not lead to the diagnosis of KHE just through clinical history and examination. Differential diagnoses of such lesions include infantile hemangioma, congenital hemangiomas that include rapidly involuting congenital hemangioma (RICH), and kaposiform lymphangiomatosis (5, 8, 9).

Hematological investigations reveal thrombocytopenia and consumptive coagulopathy in cases suggestive of KMP. KMP is a rare, life-threatening condition that occurs in 70% of patients diagnosed with KHE (10). Thrombocytopenia in KMP is usually severe, with a median platelet count of 21 × 10^9^/L (11). In this case, the infant presented with thrombocytopenia of 8 × 10^9^/L, which could likely cause severe bleeding within the compartment of her right upper limb, leading to anemia and consumptive coagulopathy. The postulation behind the thrombocytopenia has been attributed to platelet trapping within the lesion due to extracellular damage or alteration in KHE, leading to activation and aggregation of platelets, which results in subsequent consumptive coagulopathy (5). In KHE, the thrombi in the microvasculature cause vessel occlusion and prevent normal blood flow. This could lead to increased shear stress and induce increased platelet activation. This process, in turn, causes further platelet trapping and activation during the active phase of KHE (5). Given the life-threatening nature of thrombocytopenia and consumptive coagulopathy, we treated the patient with a regime of blood products to stabilize her condition upon admission as she bled from the peripheral line insertion site. The utilization of blood product support is crucial during active bleeding despite a potential worsening of KMP, with platelet transfusion aggravating platelet aggregation. In cases of active bleeding, severe coagulopathy, and/or thrombocytopenia, fresh frozen plasma and/or cryoprecipitate can be used in the treatment of KMP (12).

Imaging modalities such as ultrasound and MRI are helpful to aid the diagnosis of KHE. Ultrasound is recommended in superficial and small lesions, while MRI is helpful to diagnose deep infiltrating KHE, which may not be apparent on physical examination (13). The features suggestive of KHE on ultrasound and MRI were discussed above. A biopsy of the lesion remains the gold standard in the diagnosis of KHE and it should be performed when the patient's condition allows it and when it is considered safe (5). The infant underwent a percutaneous ultrasound doppler–guided biopsy, which was a closed biopsy under sedation performed by an experienced pediatric radiologist. A confirmatory diagnosis was crucial to differentiate KHE from other vascular abnormalities and could predict the long-term outcome and benefits of the subject therapy. The hallmark features of KHE are the infiltrating, defined, rounded, and confluent nodules that are composed of spindle endothelial cells. These cells form a malformed lymphatic channel and slit-like vascular lumen. The lumen contents include erythrocytes, platelet thrombi, eosinophilic hyaline bodies, and hemosiderin deposits (5). Immunohistochemical staining of KHE includes positivity for CD31 and CD34, which are the vascular endothelial markers that were present in this case (14).

The heterogeneity and the presence of disease-related comorbidities make the management of KHE challenging. The optimum treatment for KHE with KMP has not been established, and to date, there are no medications approved by the FDA for this condition. Ideal therapies for KHE with KMP would target cellular pathways important in abnormal vascular proliferation and growth (2). A treatment used previously includes systemic pharmacotherapy such as vincristine, corticosteroids, sirolimus, topical sirolimus or tacrolimus, ticlopidine, and aspirin (5). Multiple treatment regimens have been used for KHE with KMP with varying success, and the most recent randomized clinical trials reported encouraging results of using combination therapy with sirolimus plus prednisolone (5, 12, 15). Sirolimus, an inhibitor of the mammalian target of rapamycin (mTor), has been demonstrated to show satisfactory efficacy as oral administration in the treatment of KHE (16–19). There are a number of published reports on the use of sirolimus monotherapy in KHE with KMP, and it appears to be effective and safe in patients with life-threatening vascular anomalies (18, 20). The pathophysiology of KHE involves dysregulation of both angiogenesis and lymphangiogenesis. The classic spindle cell morphology found in KHE is thought to have a significant lymphatic component since these lesions stain with lymphatic endothelial markers D2-40 (podoplanin) and Prox-1 (21, 22). Sirolimus is effective on the lymphangiogenesis pathway, in which ligand-binding-induced signaling through VEGFR-3 on the surface of the lymphatic endothelium results in the activation of the PI3K/Akt/mTOR pathway (23, 24). The duration of treatment with sirolimus for KHE has been reported to be variable, and in a case series, it was reported that 12 months of treatment with sirolimus was associated with no disease recurrence. However, more studies are needed to investigate the most feasible duration of therapy required. Our experience suggests that sirolimus monotherapy of 12 months is a reasonable treatment for an infant with KHE with KMP, as it showed a good resolution of the lesion over the right upper limb and coagulopathy. We also monitored the patient for possible adverse effects of sirolimus including bone marrow suppression, metabolic derangements of hypercholesterolemia and hypertriglyceridemia, gastrointestinal side effects, as well as the rare side effect of pneumonia (14, 25). The patient tolerated the oral sirolimus well with only transient hypertriglyceridemia. The patient has been on a multidisciplinary team follow-up for 3 years now and has remained in continuous remission.

## Conclusion

Kaposi hemangioendothelioma with KMP is a rare vascular neoplasm that can pose a diagnostic challenge to many treating physicians. A delay in diagnosis may lead to a potentially devastating outcome for the patient. Early diagnosis and initiation of treatment promise a good outcome.

## Data Availability

The original contributions presented in the study are included in the article/Supplementary Material, further inquiries can be directed to the corresponding author/s.
